# Impact of histone deacetylase 1 and metastasis-associated gene 1 expression in esophageal carcinogenesis

**DOI:** 10.3892/ol.2014.2176

**Published:** 2014-05-26

**Authors:** TOMOHARU MIYASHITA, HIDEHIRO TAJIMA, MASAYOSHI MUNEMOTO, FURHAWN A. SHAH, JOHN W. HARMON, TOSHIFUMI WATANABE, MASATOSHI SHOJI, KOICHI OKAMOTO, SHINICHI NAKANUMA, SEISHO SAKAI, JUN KINOSHITA, ISAMU MAKINO, KEISHI NAKAMURA, HIRONORI HAYASHI, KATSUNOBU OYAMA, MASAFUMI INOKUCHI, HISATOSHI NAKAGAWARA, HIROYUKI TAKAMURA, ITASU NINOMIYA, HIROHISA KITAGAWA, SACHIO FUSHIDA, KENICHI MUKAISHO, TAKASHI FUJIMURA, TETSUO OHTA

**Affiliations:** 1 Department of Gastroenterological Surgery, Kanazawa University Hospital, Kanazawa, Ishikawa 920-8641, Japan; 2 Department of Surgery, Johns Hopkins Bayview Medical Center, Johns Hopkins University School of Medicine, Baltimore, MD 21224, USA; 3 Department of Pathology, Shiga University of Medical Science, Otsu, Shiga 520-2192, Japan

**Keywords:** metastasis-associated gene, histone deacetylases, esophageal carcinogenesis, esophageal adenocarcinoma, Barrett’s esophagus

## Abstract

Animal models are important for the development of novel therapies for esophageal cancer. Histone deacetylase 1 (*HDAC1*)/metastasis-associated gene (*MTA1*) complexes inhibit p53 acetylation and thus, inhibit p53-induced apoptosis. The aim of the present study was to evaluate HDAC1 and MTA1 expression in esophageal carcinogenesis in rats. The rats underwent a total gastrectomy followed by esophagojejunostomy to induce chronic duodenal content reflux esophagitis. The rats were sacrificed sequentially at 20, 30, 40 and 50 weeks post-surgery and the esophagi were examined. Immunohistochemical analysis was conducted to assess the expression and localization of HDAC1 and MTA1. At 20 weeks post-surgery, squamous proliferative hyperplasia and Barrett’s metaplasia (BM) were observed. While, adenocarcinoma-associated BM and squamous cell carcinoma were observed at 30–50 weeks post-surgery. The nuclear expression of HDAC1 and MTA1 was observed in all of the stages of squamous carcinogenesis and adenocarcinogenesis, although not in the normal esophageal epithelium. The expression of HDAC1 and MTA1 may be involved in duodenoesophageal reflux-induced neoplastic transformation of the esophageal mucosa into cancer cells with squamous and adeno differentiation.

## Introduction

Esophageal squamous cell carcinoma (ESCC) is the dominant type of esophageal cancer worldwide (1). However, the incidence of esophageal adenocarcinoma (EADC) has been rapidly increasing in the Western world over the last 50 years, particularly in western males (2,3). The etiology of the increase in the incidence of EADC remains obscure and has prompted further investigation into this clinical issue.

The rapid increase of EADC in Western countries has occurred in parallel with an increased prevalence of gastroesophageal reflux disease (GERD) (4,5). Diet and lifestyle alterations in the Western world have been associated with an increased prevalence of obesity and hiatal hernias, which are known risk factors for GERD and esophageal cancer (5,6). A previous study proposed that EADC develops via a sequence of events into GERD (7). Specifically, gastroduodenal content reflux from GERD induces inflammation-mediated hyperplasia and metaplasia, and subsequently dysplasia and EADC.

Studies have also determined that duodeno-esophageal or duodeno-gastro-esophageal reflux induces the sequential development of EADC in surgical rat models (8–10). These cancerous changes occur without the use of any exogenous carcinogens. The rat model demonstrated the histopathological sequence of events from GERD to EADC as an inflammation-metaplasia-dysplasia-adenocarcinoma (ADC) sequence. Furthermore, a recent study has established a correlation between the quantity of reflux and the likelihood of developing EADC and ESCC (11).

The pathogenesis of reflux-induced duodenoesophageal carcinoma and the molecular changes in gene expression, which drives esophageal carcinogenesis in rats, have recently been addressed (12). The potential role of histone deacetylase (HDAC) 1 and metastasis-associated gene (MTA) 1 in esophageal carcinogenesis remains unclear and has not been investigated in depth.

*MTA* is a newly discovered family of cancer progression-associated genes and their encoded products (13). The expression of *MTA1* and its encoded protein, MTA1, have been found to correlate with the malignant properties of numerous human cancers, including cancer of the esophagus (14), breast (15), pancreas (16), colon (17), stomach (18), liver (19) and prostate (20).

Histone acetyltransferase (HAT)- and HDAC-induced alterations of the chromatin structure have been implicated in the regulation of gene transcription, as well as in the process of carcinogenesis (21,22).

Chromatin histone and non-histone proteins are the protein targets for HDAC deacetylation via nucleosome remodeling and histone deacetylation (NuRD) complexes containing MTA proteins. The p53 tumor suppressor protein was the first non-histone protein reported to be deacetylated by MTA protein-containing NuRD complexes (23). The HDAC1/MTA1 complexes exert deacetylation activity against p53 protein in human non-small cell carcinoma and human hepatoma cells. In addition, the complexes have been found to inhibit p53-induced apoptosis by attenuating the transactivation function of p53 (18,24).

To improve the understanding of esophageal carcinogenesis in humans, animal models mimicking this tumorigenic process are particularly powerful tools. The use of experimental animal models is an effective method to understand the developmental mechanisms underlying carcinogenesis. The current study utilized a surgically induced rat reflux model of esophageal carcinogenesis. The rat surgical reflux model provided the opportunity to record the expression of proteins encoded by the *HDAC1* and *MTA1* genes in each stage of carcinogenesis, and to observe the effects on cell proliferation and carcinogenesis. In addition, the model was advantageous as it enabled examination of the expression of the *HDAC1* and *MTA1* genes in all stages of esophageal carcinogenesis, including squamous hyperplasia, squamous dysplasia, squamous cell carcinoma (SCC), Barrett’s esophagus, ADC and adenosquamous carcinoma (ASC). By improving the understanding of the expression of HDAC1 and MTA1 in esophageal carcinogenesis, targeted esophageal cancer chemotherapy may be developed.

The aim of the present study was to assess HDAC1 and MTA1 expression in a surgical rat model of esophageal carcinogenesis.

## Materials and methods

### Experimental animals

In total, 50 Wistar male rats, weighing ~250 g, were used in the present study. The animals were housed three per cage and maintained at a constant room temperature of 22±3°C, in 55±5% humidity under a 12-h light-dark cycle. The rats were fed standard solid chow (CRF-1; Charles River Laboratories Japan, Inc., Yokohama, Japan) and tap water that was free of carcinogens. The study was approved by the Institutional Animal Care and Use Committee of the Graduate School of Medical Science, Kanazawa University (AP-111868; Kanazawa, Japan).

### Surgical procedures

Following a 24-h fast, an upper abdominal incision was made under diethyl ether inhalation anesthesia. The surgical procedures were performed to induce duodenoesophageal reflux following total gastrectomy of each rat as previously reported (10).

### Specimen extraction

The animals were sacrificed by diethyl ether inhalation and the abdomen was opened. A ligature was placed around the afferent and efferent jejunal loop proximal to the esophagojejunal anastomosis. The esophagus was ligated at the level of the thyroid cartilage via a thoracotomy. The esophagus and anastomosed jejunum were subsequently removed.

### Pathological assessment

The excised organs were washed with 10% formalin, spread and pinned on a cork plate with the mucosal side facing upwards. Following fixation of the organs with 10% formalin solution for at least 24 h, the esophagus was cut into slices along the longitudinal axis at 3-mm intervals and embedded in paraffin. Next, 5 μm-thick sections of each embedded paraffin block were prepared for histological analysis with hematoxylin and eosin staining.

### Immunohistochemistry

For the immunohistochemical staining, the Dako Envision system (Dako, Carpinteria, CA, USA), which uses dextran polymers conjugated with horseradish peroxidase, was employed to avoid any endogenous biotin contamination. The sections were deparaffinized in xylene and rehydrated in a graded ethanol series. The endogenous peroxidase was blocked by immersing the sections in 3% H_2_O_2_ in 100% methanol for 20 min at room temperature. Antigen retrieval was achieved by microwaving the sections at 95°C for 10 min in 0.001 M citrate buffer (pH 6.7). After blocking the endogenous peroxidase, the sections were incubated with Protein Block Serum-Free (Dako) at room temperature for 10 min to block the non-specific staining. The sections were incubated for 2 h at room temperature with 1:100 diluted mouse antibodies against monoclonal MTA1 (D40D1; Cell Signaling Technology, Inc., Beverly, MA, USA) and polyclonal HDAC1 (ab19845; Abcam, Cambridge, MA, USA). Peroxidase activity was detected with the enzyme substrate, 3-amino-9-ethylcarbazole and for the negative controls, the sections were incubated with Tris-buffered saline (Wako Pure Chemical Industries, Tokyo, Japan) without the primary antibodies. The samples with ≥10% of tumor cells were slightly counterstained with Mayer hematoxylin and considered to show positive staining. Immunohistochemical staining for MTA1/HDAC1 was scored by two of the authors as positive or negative in the epithelium that was under examination. An estimation of the immunohistochemical expression of the markers was determined by counting ≥100 cells in random high-power fields. The frequency of positive cells for each antibody was reported semi-quantitatively as follows: (−), No reaction; (+), mild with <30% of positive cells; (++), moderate with 30–60% of positive cells; and (+++), marked with >60% of positive cells. A positive expression was defined as the staining of >30% of the cancer cells (++ or +++).

### Definition of pathological findings

The histological findings in the esophagus were classified into the following four categories: i) Proliferative squamous hyperplasia (PHP), a condition characterized by a thickened epithelium to twice that of a normal epithelium, with acanthosis, elongation of the papillae and parakeratosis, as well as thickening of the basal layer of the squamous epithelium and preservation of a stratified appearance; ii) squamous dysplasia, characterized by an epithelium composed of dysplastic squamous cells with large and polymorphic nuclei with deeply stained chromatin and an increased number of mitotic figures, which may involve the lamina propria of the epithelium, however, do not invade the submucosal layer; iii) Barrett’s metaplasia (BM), presents replacement of the esophageal squamous epithelium with columnar-lined epithelium comprised of gastric and/or intestinal cells; and iv) carcinoma, defined as cellular and structural atypism with epithelial invasion into the submucosal layer. ADC consists of dysplastic glandular cell growth, with atypia and invasiveness, and exhibits two types of histology: Tubular or papillary ADC; and mucinous ADC. SCC is a type of squamous cell dysplasia with marked cellular and structural atypism, which may be divided into well- and poorly-differentiated types according to the presence or absence of cancer pearls, respectively.

## Results

### Histological findings

**Figure 1:**
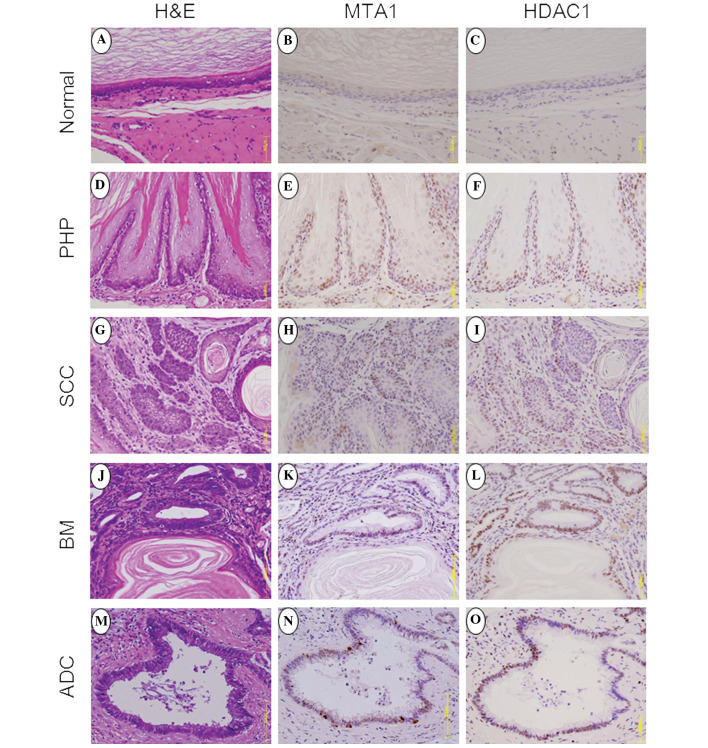
(A) Normal squamous epithelium did not stain for (B) MTA1 or (C) HDAC1. (D) PHP showing papillary projections and hyperkeratosis with (E) MTA1 and (F) HDAC1 expression in the two to three basal layers of papillary hyperplasia. (G) Pure SCC showing dysplastic squamous cells with marked structural atypism and cancer pearls were stained positive for (H) MTA1 and (I) HDAC1. (J) BM surrounded by a squamous lesion expressing (K) MTA1 and (L) HDAC1 in the nucleus. (M) Pure ADC showing dysplastic glandular cell growth with atypia and invasiveness stained positive for (N) MTA1 and (O) HDAC1 expression in the nucleus. H&E, hematoxylin and eosin; MTA, metastasis-associated gene; HDAC, histone deacetylase; PHP, proliferative squamous hyperplasia; SCC, squamous cell carcinoma; BM, Barrett’s metaplasia; ADC, adenocarcinoma.

In total, 40/50 rats survived following the surgery and were used in the present study. Immunohistochemistry was performed (Fig. 1) and demonstrated normal esophageal epithelium in the upper esophagus at 20 weeks post-surgery (Fig. 1A), as well as squamous PHP (Fig. 1D), dysplasia and BM. At 30–50 weeks following surgery, 13/35 (37%) rats had developed esophageal cancer. In addition, SCC (Fig. 1G) was observed in 4/35 (11%) of the rats. By contrast, dysplastic changes, including BM (Fig. 1J) and ADC (Fig. 1M), were observed within 30 weeks and increased sequentially to 100 and 40%, respectively at 40–50 weeks (Table I).

### MTA1 expression

**Figure 2:**
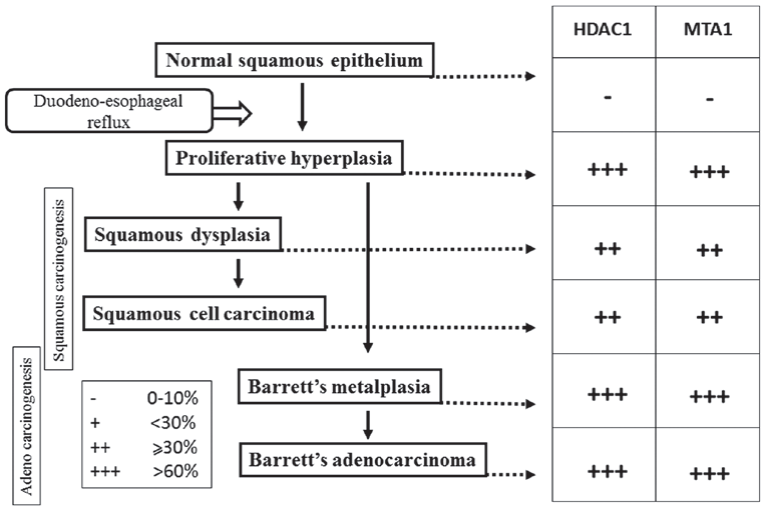
Immunohistochemical pattern of MTA1 and HDAC1 expression through the progression from normal epithelium to different types of carcinoma. HDAC1, histone deacetylase 1; MTA1, metastasis-associated gene 1.

A high positive expression of MTA1 was identified in the basal layer of PHP (Fig. 1E), but not in the normal epithelium at 20 weeks (Fig. 1B). At 30 weeks, BM showed a high positive expression of MTA1 (Fig. 1K). ADC-associated BM showed a high positive expression of MTA1 at 30–50 weeks post-surgery (Fig. 1N). By contrast, SCC demonstrated marginally reduced expression of MTA1 at 30–50 weeks post-surgery (Fig. 1H). The MTA1 immunohistochemistry staining showed nuclear expression of MTA1 in all of the stages of squamous carcinogenesis, including PHP, squamous dysplasia and SCC, and adenocarcinogenesis, including BM and ADC, however, this was not observed in the normal squamous epithelium (Fig. 2).

### HDAC1 expression

Similarly, the positive expression of HDAC1 was found in PHP (Fig. 1F), BM (Fig. 1L), ADC (Fig. 1O) and SCC (Fig. 1I), but not identified in the normal squamous epithelium (Fig. 1C). Furthermore, the HDAC1 immunohistochemical staining showed nuclear expression of HDAC1 in all of the stages of squamous carcinogenesis, including PHP, squamous dysplasia and SCC, and adenocarcinogenesis, including BM and ADC, however, this was not observed in the normal squamous epithelium (Fig. 2).

## Discussion

The incidence of GERD-induced esophageal carcinoma is rising in the USA and the Western world (4,5). We have pioneered the use of a rat reflux model of esophageal carcinoma, which is based on surgically inducing duodenogastroesophageal reflux akin to GERD in humans without the use of a carcinogen (8–10). The model has been successfully used to investigate reflux-induced esophageal carcinogenesis. While the correlation between reflux and esophageal carcinoma has been investigated in a number of studies, the molecular mechanisms underlying esophageal carcinogenesis remains poorly understood (8–10).

Numerous molecular alterations leading to the development of esophageal carcinoma have been reported (12). Chronically inflamed tissue results in the activation of multiple signaling pathways that lead to inflammation and tumorigenesis. A number of these factors, such as NF-κB and Egr-1, have been shown to have additive or synergistic effects on the activation of a number of inflammation-associated genes, particularly those that are associated with the neoplastic transformation (25).

The alterations of the chromatin structure by *HATs* and *HDACs* are implicated in the regulation of gene transcription, as well as in the process of carcinogenesis. Tumors demonstrate the hyperacetylation of histone H4 and the increased expression of *HDAC1*, thus implying that a certain interaction may exist between the hyperacetylation of histone H4 and *HDAC1* expression (21). ΔNp63α, an N-terminally truncated form, which functions as a key ESCC cell survival factor, associates with *HDAC1* and *HDAC2* to form an active transcriptional repressor complex that may be targeted to provide a therapeutic advantage. The repression of the proapoptotic Bcl-2 family member genes, including *p53* upregulated modulator of apoptosis, by *p63*/*HDAC* is required for the survival of ESCC cells (26). Therefore, the immunohistochemical determination of HDACs may aid with predicting the response to specific HDAC inhibitors (27).

*MTA* is a newly discovered family of cancer progression-associated genes (13). *MTA1*, the first gene identified in this family, has been repeatedly reported to be overexpressed along with its protein product, MTA1, in a wide range of human cancers. Therefore, MTA1 may be one of the significant molecules in cancer progression. Esophageal cancers have been investigated for *MTA1/*MTA1 overexpression and those esophageal cancer cells that were overexpressing *MTA1*/MTA1 have shown significantly higher frequencies of adventitial invasion and lymph node metastasis, as well as higher rates of lymphatic involvement (28). Thus, the MTA1 protein may be a useful predictor of the malignant potential of ESCC (14).

ATP-dependent chromatin-remodeling complexes open chromatin structures and facilitate transcriptional activation. A novel human complex, NURD, contains ATP-dependent nucleosome remodeling activity and HDAC activity that associate with transcriptional repression. The NuRD complexes share four core proteins (HDAC1, HDAC2, RbAp46 and RbAp48) with the Sin3 complex. These complexes contain the dermatomyositis-specific autoantigens, Mi-2α/β, MTA1/2 and p66, which are functionally and physically linked (29–33).

The MTA1 protein, a component of the NuRD complex, possesses strong transcription-repressing activity (30). MTA2, another component of the NuRD complex, is highly expressed in rapidly dividing cells (32). Toh *et al* (34) reported the physical interaction between the MTA1 protein and HDAC1. The MTA protein family members basic functions of chromatin remodeling and histone deacetylating activities are exerted via NuRD complexes. While there are additional non-histone deacetylating proteins in NuRD complexes, the MTA proteins are likely to be the principal components. In addition, the MTA-NuRD complexes show transcription-repression activities (35,36). Although all MTA protein family members are found in NuRD complexes, each MTA protein may form a distinct NuRD complex that targets different sets of promoters (33). Thus, the MTA-HDAC complex is further involved in the normal transcriptional balance of the cell (37).

HDAC, via NuRD complexes containing MTA proteins, deacetylates, chromatin histones and non-histone proteins. The p53 tumor suppressor protein was the first non-histone protein reported to be deacetylated by MTA protein-containing NuRD complexes (23,37). A HDAC1 complex, which contains the MTA2 protein mediates the deacetylation of p53. An MTA2-associated NuRD complex is also involved, and this HDAC1/MTA2 complex interacts with p53 *in vitro* and *in vivo*, which significantly reduces the steady-state levels of acetylated p53. The deacetylation of p53 causes an increase in its own degradation through MDM2 and a reduction in p53-dependent transcriptional activation. Eventually, this results in the repression of the normal p53 function that mediates cell growth arrest and apoptosis (23,38). The same phenomenon is observed between the p53 and MTA1 complex (23,24). Previous studies have determined that the HDAC1/MTA1 complexes deacetylate the p53 protein and attenuate the transactivation function of p53 in human carcinoma, thereby inhibiting p53-induced apoptosis (18,24). The MTA proteins have been shown to be ubiquitinated transcriptional corepressors, which function in HDAC and are part of the NuRD complex, a nucleosome remodeling and HDAC complex whose stability appears to be regulated by the binding of ubiquitinated MTA1 to E3 ubiquitin ligase constitutive photomorphogenesis protein-1 (37,39). The MTA1 protein inhibits p53-induced apoptosis by deacetylation of p53, which may be associated with the increased metastatic potential of cancer cells with high MTA1 expression (23,24).

Miyatani *et al* (40) examined a possible association between HDAC1 and MTA1 expression, and disease progression in the esophageal metaplasia-dysplasia-carcinoma sequence and particularly in low-grade dysplasia. The percentage of HDAC1- and MTA1-positive expression in low-grade dysplasia of BM was as high as that of cancer. Therefore, BM with increased HDAC1 and MTA1 expression is considered to be a precancerous lesion. The results of the current study showed that MTA1 and HDAC1 expression is already present in PHP and BM. The positive expression rate of MTA1 and HDAC1 was similar to that among PHP, BM and EADC. Thus, increased MTA1 and HDAC1 expression indicates that PHP and BM may already have malignant potential.

Bonde *et al* (12) established that cell lines derived from a reflux-associated rat model of esophageal cancer exhibited glandular features. While the histology of the xenografts expressed a squamous phenotype when they were transplanted into nude mice. Cytogenetic analysis also showed significant similarities between rat and human esophageal cancers, including ESCC and EADC. Furthermore, cytogenetic analysis of the rat model revealed a highly aneuploid cell population with the derangement of key chromosomes that encode a variety of oncogenes. The neoplastic nature, gene expression profile and pathway activation of these rodent reflux-induced tumors was found to be comparable with human esophageal cancer. In rats with BM and EADC, the deletion and translocation of chromosome 7 and 11, and overexpression of important peptide mediators are considered to be significant in carcinogenesis, including hypoxia-inducible factor (HIF) 1α, cyclin dependent kinase 4, vascular endothelial growth factor, polo-like kinase and the epidermal growth factor receptor.

HIF1α is a key regulator of angiogenic factors (41) and another important non-histone protein that is deacetylated by the HDAC1/MTA1 complexes. The MTA1 protein binds to and deacetylates HIF1α, which increases HDAC1 expression and subsequently stabilizes HIF1α via a positive feedback mechanism (23). The expression of HDAC1 and MTA1 is similar in squamous carcinogenesis and adenocarcinogenesis. Therefore, esophageal squamous and adeno differentiation may possess a common genetic pathway.

Histologically, the spectrum of esophageal cancer is divided into ESCC and EADC. However, the mechanism by which each histological type of carcinoma arises from the esophageal mucosa remains unknown. In our previous study, the rodents received one of the following procedures: Duodeno-forestomach reflux with reduced exposure to duodenal contents; duodenoesophageal reflux with increased exposure to duodenal contents; or three control operations. The fraction of ESCC in the reduced exposure group was significantly higher than that of the high exposure group, while the fraction of EADC in the reduced exposure group was significantly lower than that of the high exposure group. In brief, high exposure to duodenal contents promotes the development of EADC, and low exposure induces ESCC. The severity of the duodenoesophageal reflux in rodents is associated with the development of different histological types of esophageal carcinoma (11). In the present study, ESCC was shown to arise from dysplastic changes to squamous cells that were naturally located proximal to the columnar-lined epithelium. By contrast, EADC arose in the areas of columnar-lined epithelium adjacent to the surgical anastomosis.

MTA1 expression levels are closely associated with the degree of malignant potential. For example, metastatic malignant tissue demonstrates increased expression when compared with tissue from high-grade prostatic intraepithelial neoplasia (42). In the present study, squamous PHP was found to express MTA1 and HDAC1 and therefore, PHP may have malignant potential, which chemopreventive agents could be directed against.

The present study was not designed to completely evaluate the esophageal cancer signaling pathway. However, the results are consistent with those of previous studies, which demonstrate that the *MTA1/HDAC1* complexes are involved in other malignancies. This study also demonstrates the involvement of these complexes in esophageal cancer.

Thus, it has been proposed that the MTA and HDAC proteins, particularly MTA1 and HDAC1, present as master coregulatory molecules involved in esophageal carcinogenesis.

The HDAC inhibitors have been shown to be potent inducers of growth arrest, differentiation and/or apoptotic cell death of transformed cells and, as a result, are currently receiving considerable attention as antitumor agents. In addition, the HDAC inhibitors have been reported to disrupt the cell cycle in the G2 phase, allowing cells to prematurely enter the M phase, as well as to interfere directly with the mitotic spindle checkpoint (43).

Kai *et al* (20) reported a novel pathway of chromatin remodeling by resveratrol (Res) via the functional restriction of the MTA1/NuRD complex; Res decreased MTA1 expression, thus deregulating the MTA1*/*HDAC1 complexes leading to increased p53 acetylation (Ac-p53), and enhanced binding to the p21 and Bax promoters in the PCa cells. Furthermore, Res treatment on MTA1-null background resulted in a marked increase in the apoptotic function of Res, which indicates MTA1 as an antiapoptotic protein. Finally, the combined treatment of Res and the HDAC inhibitor, SAHA, cooperatively increased Ac-p53 levels and apoptosis, indicating novel therapeutic strategies in combination with the use of HDAC inhibitors that have already been clinically approved (20). Thus it is necessary to administer the HDAC inhibitor and Res for the treatment of GERD patients at an early stage.

In conclusion, the expression of HDAC1 and MTA1 may be required to promote the development of neoplastic transformation in cancer cells with squamous and adeno differentiation. Furthermore, the HDAC inhibitor and administration of Res may prevent esophageal carcinogenesis.

## Figures and Tables

**Table I:** Outcome and histological findings.

	Postoperative week			
	
	20	30	40	50
Rats examined, n	5	10	10	15
Histology, n (%)				
Proliferative hyperplasia	5 (100)	10 (100)	10 (100)	15 (100)
Squamous dysplasia	1 (20)	5 (50)	6 (60)	6 (40)
Squamous cell carcinoma	0 (0)	1 (10)	1 (10)^a^	2 (13)^a^
Barrett’s metaplasia	2 (40)	7 (70)	10 (100)	15 (100)
Adenocarcinoma	0 (0)	1(10)	4 (40)	6 (40)

a One rat exhibited two types of carcinoma.

## Abbreviations

ESCC: esophageal squamous cell carcinoma
EADC: esophageal adenocarcinoma
HDAC: histone deacetylases
MTA: metastasis-associated gene
GERD: gastroesophageal reflux disease
